# Supplementary material for: Understanding the Influence of a Bifunctional Polyethylene Glycol Derivative in Protein Corona Formation around Iron Oxide Nanoparticles
Source: Materials (Basel). 2019 Jul 10;12(14):2218. doi: 10.3390/ma12142218 (PMC6678275; doi:10.3390/ma12142218)
Supplement: Supplementary file 1 [file materials-12-02218-s001.pdf]

materials

Supplementary Materials

Understanding the Influence of a Bifunctional Polyethylene Glycol Derivative in Protein Corona Formation around Iron Oxide Nanoparticles

Amalia Ruiz [1,*,†], Adán Alpízar [2], Lilianne Beola [3], Carmen Rubio [4], Helena Gavilán [5], Marzia Marciello [5,6], Ildefonso Rodríguez-Ramiro [7], Sergio Ciordia [2], Christopher J. Morris [1] and María del Puerto Morales [5,*]

1 School of Pharmacy, University of East Anglia, Norwich Research Park, NR4 7TJ Norwich, UK.
2 Centro Nacional de Biotecnología (CNB)/CSIC, Darwin, 3, 28049 Madrid, Spain.
3 Instituto de Ciencia de Materiales de Aragón (ICMA), CSIC/Universidad de Zaragoza, C/Pedro Cerbuna 12, 50009 Zaragoza, Spain.
4 Centro de Biología Molecular "Severo Ochoa" (CBMSO)/UAM-CSIC, Nicolás Cabrera, 1, 28049 Madrid, Spain.
5 Instituto de Ciencia de Materiales de Madrid (ICMM)/CSIC, Sor Juana Inés de la Cruz 3, Cantoblanco, 28049 Madrid, Spain
6 Faculty of Pharmacy, Complutense University of Madrid (UCM), Plaza Ramón y Cajal s/n, 28040 Madrid, Spain.
7 School of Medicine, University of East Anglia, Norwich Research Park, NR4 7TJ Norwich, UK
* Correspondence: G.RuizEstrada@qub.ac.uk (A.R.); puerto@icmm.csic.es (M.P.M.)
† Present address: School of Pharmacy, Queen’s University Belfast, 97 Lisburn Rd, Belfast BT9 7BL, UK

materials

MDPI

Table S1. Proteins identified in the soft corona of the nanoparticles after the elution with buffer 50 mM HEPES 0.1% OGP, although proteins detected with only one peptide have to be considered at t the limits of reliability.

Protein	NP-DMSA			NP-PEG-$(NH_2)_{2(2000)}$		
	Score[1]	Num. Pept[2]	Coverage[3]	Score[1]	Num. Pept[2]	Coverage[3]
Complement C3	6727.39	109	63.7	8206.05	125	67
Alpha-1-inhibitor 3	7953.42	113	54	8981.66	125	62.6
Murinoglobulin-1	7468.93	106	55.1	8099.95	114	59.1
Alpha-1-macroglobulin	7077.54	105	69.3	7902.6	113	70.5
Serum albumin	5344.66	83	78.9	6766.94	102	84.2
Murinoglobulin-2	5215.35	75	38.7	6038.86	87	49.4
Complement C4	3914.24	65	52.6	5030.55	77	55.8
Serotransferrin	3426.83	52	61.7	4157.15	61	62.6
Ceruloplasmin	2711.08	46	48.4	2903.29	48	56.8
Serine protease inhibitor A3L	2226.83	34	67.6	2713.15	37	67.1
Hemopexin	2094.57	35	60.2	2394.4	36	60.2
Alpha-1-antiproteinase	1889.01	29	53.3	2233.93	33	55.5
Plasminogen	1430.96	24	37.6	2007.56	33	40.8
Serine protease inhibitor A3K	2195.32	32	58.9	2436.74	32	58.7
Apolipoprotein A-I	1869.59	31	69.5	2023.03	30	70.3
Ig gamma-2A chain C region	2004.15	32	74.8	2072.88	30	78
Apolipoprotein A-IV	1679.47	25	70.6	1825.51	29	75.4
Vitamin D-binding protein	1771.7	25	45.4	1991.24	28	45.6
Serine protease inhibitor A3N	1464.47	23	52.9	1917.66	28	62

Afamin	1339.87	24	44.4	1549.1	27	44.1
Inter-alpha-trypsin inhibitor heavy chain H3	1432.98	24	26.8	1825.36	27	33.4
Gelsolin	932.96	17	27.8	1723.05	27	40.4
Fibronectin	1121.64	23	14.9	1532.53	26	17.4
Prothrombin	1230.14	22	41.8	1701.54	26	45.1
Apolipoprotein E	1379.82	21	59.9	1696.13	25	60.9
Apolipoprotein B-100	332.13	8	2.5	963.22	25	7.5
T-kininogen 1	1484.3	22	41.4	1603.63	24	41.4
Haptoglobin	1408.39	24	48.1	1501.84	24	48.1
T-kininogen 2	1262.76	20	45.8	1381.26	23	47.7
Plasma kallikrein	1055.78	18	33.4	1274.59	22	39.5
Complement component C9	1041.6	19	35.7	1182.51	21	37.2
Plasma protease C1 inhibitor	1155.63	16	39.1	1464.75	21	50
Histidine-rich glycoprotein	1065.86	19	44	1309.38	21	45.5
Ig gamma-1 chain C region	866.29	14	36.8	1269.67	20	62.6
Hemoglobin subunit beta-1	1153.46	19	85.7	1230.29	18	84.4
Kininogen-1	984.8	16	25.4	1198.93	18	28.2
Carboxylesterase 1C	1042.31	16	43	1250.95	18	43
Hemoglobin subunit beta-2	1090.93	18	84.4	1139.65	16	83
Serine protease inhibitor A3M (Fragment)	1090.65	17	50.5	1032.07	16	43
Alpha-2-HS-glycoprotein	1138.08	15	61.1	1104.31	15	48.3
Clusterin	666.28	12	30.6	906.66	14	29.3
Ig kappa chain C region, B allele	862.28	11	81.1	994.1	12	73.6
Hemoglobin subunit alpha-1/2	848.57	12	66.2	893.73	12	66.2
Fetuin-B	686.33	11	31	759.43	12	23.5
Transthyretin	748.29	11	67.3	812.26	11	71.4

Ig gamma-2B chain C region	749.34	12	55.3	873.5	11	40.8
C4b-binding protein alpha chain	405.66	10	21.5	469.41	11	22.4
Heparin cofactor 2	507.02	9	23.2	614.39	11	23.6
Complement factor I	478.84	9	20.7	594.73	11	20.2
Angiotensinogen	449.63	7	23.7	694.43	10	31.9
C-reactive protein	396.17	5	21.7	692.32	10	55.7
Serum paraoxonase/arylesterase 1	495.13	8	37.5	687.66	10	40.6
Complement component C8 beta chain	255.34	6	11	422.12	10	19.7
Keratin, type I cytoskeletal 10	314.38	6	9.1	566.77	10	15
Ig kappa chain C region, A allele	562.59	8	44.3	617.73	9	44.3
Serum amyloid P-component	382.18	7	44.3	667.05	9	47.8
Beta-2-glycoprotein 1	420.97	7	24.6	492.87	9	37.4
Corticosteroid-binding globulin	609.09	10	24.5	533.91	9	24.5
Protein Z-dependent protease inhibitor	220.86	3	12.4	605.49	9	33.3
Phosphatidylinositol-glycan-specific phospholipase D	406.83	7	10.9	432.8	9	15.4
Coagulation factor XII	221.91	4	10.4	416.96	8	16.1
Ig gamma-2C chain C	395.85	8	24.9	467.26	8	28.6
Ig lambda-2 chain C	424.97	7	72.1	454.76	7	72.1
Insulin-like growth factor-binding protein complex acid labile subunit	165.28	3	7.3	337.22	7	17.1
Keratin, type II cytoskeletal 1	205.68	4	7	444.63	7	8.3
Alpha-1-acid glycoprotein	143.53	4	12.7	287.16	6	27.8
Retinol-binding protein 4	267.81	5	34.3	335.41	6	39.3
Alpha-2-macroglobulin	276.62	4	2.1	387.51	6	3.7
Zinc-alpha-2-glycoprotein	179.41	4	23.3	297.05	6	24.3
Alpha-1B-glycoprotein	226.34	5	9.9	275.13	6	11.3

Carboxypeptidase N catalytic chain	138.35	3	10.5	215.74	6	20.6
Carboxypeptidase B2	68.73	2	9.2	289.63	6	23
Glutathione peroxidase 3	294.8	6	28.3	346.68	5	31.9
Actin, cytoplasmic 1	288.62	7	27.2	245.14	5	16.8
Protein AMBP	124.03	2	6.3	282.95	5	16.9
Apolipoprotein A-II	91.09	2	19.6	247.94	4	49
Complement factor D	71.45	1	5.7	268.46	4	25.9
Keratin, type II cytoskeletal 6A	76.21	1	2.2	210.01	4	6.9
Extracellular matrix protein 1	86.9	2	4.4	231.1	4	9.1
Coagulation factor X	81.97	2	6.6	171.47	4	10.8
Carboxypeptidase Q	58.24	1	2.3	205.27	4	11.9
Complement component C6	158.26	4	6.9	154.94	4	6.2
Major urinary protein	94.16	2	13.3	114.31	3	17.7
Beta-2-microglobulin	149.37	3	26.1	183.82	3	26.1
Phosphatidylcholine-sterol acyltransferase	23.19	1	3.9	106.27	3	7
Apolipoprotein C-I	167.53	3	22.7	189.53	3	22.7
Mannose-binding protein A	26.7	1	2.9	162.36	3	10.9
Selenoprotein P	--	--	--	125.38	3	8.6
Complement C1q subcomponent subunit A	--	--	--	89.29	3	15.9
Apolipoprotein C-IV	186.54	4	32.3	171.24	3	25
Carboxylesterase 1E	--	--	--	145.31	3	11.4
Neuronal PAS domain-containing protein 4	--	--	--	71.24	3	4.1
Leukemia inhibitory factor receptor	41.15	1	0.9	65	2	1.6
Ig heavy chain V region IR2	--	--	--	145.71	2	17.6
Glyceraldehyde-3-phosphate dehydrogenase	--	--	--	65.74	2	10.8
Platelet factor 4	108.86	2	21.9	138.46	2	21.9

Apolipoprotein M	76.03	2	14.7	54.04	2	14.7
Carbonic anhydrase 2	35.2	1	3.8	60.72	2	11.2
Vitamin K-dependent protein S	45.22	1	2.2	88.9	2	4.1
Keratin, type I cytoskeletal 15	--	--	--	104.4	2	3.6
Keratin, type II cytoskeletal 2 epidermal	28.47	1	1.3	104.27	2	3.4
Attractin	59.87	2	2	50.39	2	2.7
Embryonic polyadenylate-binding protein 2	--	--	--	20.41	1	3.7
Failed axon connections homolog	--	--	--	21.49	1	1.7
Microtubule-actin cross-linking factor 1	--	--	--	23.23	1	0.5
Serine/threonine-protein kinase 10	--	--	--	28.55	1	1.1
Cell division cycle 5-like protein	21.35	1	1.1	21.94	1	1.1
Ribonuclease 4	--	--	--	68.52	1	8.8
Cadherin EGF LAG seven-pass G-type receptor 3	--	--	--	24.68	1	0.2
Fibrinogen alpha chain	--	--	--	105.62	1	2
Receptor tyrosine-protein kinase erbB-2	21.36	1	1.1	28.11	1	1.1
Annexin A1	--	--	--	22.52	1	2
Cationic trypsin-3	56.62	1	3.2	37.1	1	3.2
Protein kinase C delta type	--	--	--	27.7	1	1.2
A-kinase anchor protein SPHKAP	22.02	1	0.5	22.5	1	0.5
cAMP-dependent protein kinase type II-alpha regulatory subunit	--	--	--	30.86	1	2.5
Myosin-3	--	--	--	21.81	1	0.5
Insulin-like growth factor-binding protein 3	--	--	--	65.57	1	2.7
Class I histocompatibility antigen, Non-RT1.A alpha-1 chain	--	--	--	20.38	1	4.1
Coagulation factor IX (Fragment)	98.95	2	6	55.39	1	3.2
Proteasome subunit alpha type-1	--	--	--	20.9	1	2.7

Band 3 anion transport protein	--	--	--	52.07	1	2
Plectin	--	--	--	20.12	1	0.2
Sodium-dependent serotonin transporter	25.36	1	1.1	21.07	1	1.1
Complement C1q subcomponent subunit B	184.8	4	18.6	88.44	1	4.7
Complement C1q subcomponent subunit C	--	--	--	58.73	1	4.1
Microtubule-associated protein 1A	--	--	--	24.72	1	0.3
Signal peptidase complex catalytic subunit SEC11A	--	--	--	23.15	1	3.9
Metalloproteinase inhibitor 3	--	--	--	68.02	1	5.7
Nuclear pore complex protein Nup153	26.64	1	0.6	23.08	1	0.6
Lumican	51.57	1	3.3	41.45	1	3.3
Inactive pancreatic lipase-related protein 1	--	--	--	27.02	1	1.5
Tropomyosin beta chain	--	--	--	34.02	1	3.9
Pannexin-1	49.1	1	1.6	32.1	1	1.6
40S ribosomal protein S13	--	--	--	20.1	1	4.6
Adenomatous polyposis coli protein	22.83	1	0.2	21.32	1	0.2
Activin receptor type-1	--	--	--	22.7	1	1.4
Lipid phosphate phosphohydrolase 3	--	--	--	20.38	1	2.2
Unconventional myosin-Ib	--	--	--	22.3	1	0.6
Extracellular superoxide dismutase [Cu-Zn]	--	--	--	74.49	1	6.6
Enoyl-CoA hydratase domain-containing protein 3, mitochondrial	37.94	1	2.3	38.2	1	2.3
Charged multivesicular body protein 5	20.9	1	4.6	21.61	1	4.6
RNA polymerase II-associated factor 1 homolog	--	--	--	23.94	1	2.6
Leucine-rich repeat-containing protein 63	29.92	1	1.2	28.19	1	1.2
Zinc finger RNA-binding protein	--	--	--	25.32	1	0.8
Zinc finger and BTB domain-containing protein 38	--	--	--	27.16	1	0.7

Biotinidase	--	--	--	34.88	1	2.5
Signal-induced proliferation-associated 1-like protein 2	22.03	1	0.4	22.31	1	0.4
Transmembrane protease serine 11G	--	--	--	21.64	1	1.7
Zinc finger protein DZIP1L	--	--	--	20.83	1	1.7
Calcium-dependent secretion activator 1	--	--	--	20.29	1	1
Non-muscle caldesmon	21.58	1	1.3	29.49	1	1.3
Dual specificity mitogen-activated protein kinase kinase 5	44.81	1	1.6	41.97	1	1.6
Eukaryotic translation initiation factor 2-alpha kinase 1	24.22	1	1.1	24.14	1	1.1
Lipopolysaccharide-binding protein	--	--	--	37.22	1	2.3
Interleukin-1 receptor accessory protein	74.55	2	4	47.7	1	2.3
Secernin-1	20.29	1	1.9	23.09	1	1.9
Signal recognition particle 54 kDa protein	--	--	--	29.01	1	1.4
Keratin, type II cytoskeletal 4	--	--	--	39.85	1	3
Complement C1s subcomponent	--	--	--	48.18	1	1
SUN domain-containing ossification factor	--	--	--	42.57	1	0.7
ATP-binding cassette sub-family A member 7	26.44	1	0.4	32.49	1	0.4
Serpin A11	--	--	--	29.26	1	2.6
Calsyntenin-3	--	--	--	20.55	1	0.8
Apoptotic protease-activating factor 1	28.14	1	0.9	26.95	1	0.9
Intraflagellar transport protein 172 homolog	30.95	1	0.7	34.41	1	0.7
Vesicle-fusing ATPase	28.5	1	1.2	25.82	1	1.2
Carbonic anhydrase 1	57.13	1	3.8	--	--	--
Cation channel sperm-associated protein subunit delta	22.87	1	1.1	--	--	--
Chromodomain-helicase-DNA-binding protein 6	52.82	2	0.3	--	--	--
Sentrin-specific protease 7	23.75	1	0.8	--	--	--
Cytochrome P450 2F2	20.27	1	2.4	--	--	--

Calpain-9	20.89	1	1.2	--	--	--
Dipeptidyl peptidase 3	23.01	1	1.2	--	--	--
Calpain-6	21.19	1	1.2	--	--	--
Apolipoprotein C-III	25.49	1	29.7	--	--	--
Complement C5 (Fragment)	37.79	1	10.4	--	--	--
Mesencephalic astrocyte-derived neurotrophic factor	20.31	1	4.5	--	--	--
60S ribosomal protein L13	22.77	1	4.3	--	--	--
Nucleolar and coiled-body phosphoprotein 1	24.08	1	1.3	--	--	--
Regulator of G-protein signaling 9	22.39	1	1.2	--	--	--
Phospholipase A-2-activating protein	28.8	1	1	--	--	--
Cerebellin-1	24.98	1	3.6	--	--	--
Adenosine deaminase domain-containing protein 1	23.02	1	1.8	--	--	--
Importin subunit alpha-6	20.07	1	1.3	--	--	--
Exocyst complex component 3	23.63	1	1.5	--	--	--
Muscle, skeletal receptor tyrosine protein kinase	21.92	1	2	--	--	--
Golgi SNAP receptor complex member 1	24.29	1	2.8	--	--	--
Gamma-glutamyltransferase 6	23.67	1	1.6	--	--	--
Elongator complex protein 1	41.85	1	0.5	--	--	--
Indoleamine 2,3-dioxygenase 1	20.56	1	2.9	--	--	--
Glyceraldehyde-3-phosphate dehydrogenase, testis-specific	47.53	1	3.2	--	--	--
Alpha-parvin	21.6	1	4	--	--	--
Regulator of G-protein signaling 2	20.78	1	6.2	--	--	--
Cyclin-L1	21.6	1	2.1	--	--	--
Structural maintenance of chromosomes protein 1A	21.55	1	0.6	--	--	--

[1] Score: number which reflects the combined scores of all observed mass spectra that can be matched to amino acid sequences within that protein. A higher score indicates a more confident match.

[2] Num. Pept: Number of peptides detected for each protein identified.

[3] Coverage: Percentage of the database protein sequence covered by matching peptide.

Table S2. Proteins identified in the hard corona of the nanoparticles after the elution with buffer 100 mM NaAc 0.1% OGP pH 5, although proteins detected with only one peptide have to be considered at t the limits of reliability.

Protein	NP-DMSA			NP-PEG-$(NH_2)_{2(2000)}$		
	Score[1]	Num. Pept[2]	Coverage[3]	Score[1]	Num. Pept[2]	Coverage[3]
Alpha-1-inhibitor 3	6811.66	104	51.2	5886.15	90	42.5
Murinoglobulin-1	6297.03	98	50.6	5203.87	81	41
Complement C3	6681.66	105	59.6	5169.88	81	44.2
Serum albumin	4960.14	77	70.6	4941.58	74	65
Alpha-1-macroglobulin	6562.77	98	59.5	4622.58	70	41.9
Serotransferrin	4210.72	63	62	4160.3	65	52.3
Plasminogen	3174.39	53	50.6	3824.04	63	52.2
Murinoglobulin-2	4323.68	71	37.8	3740.35	59	30.4
Complement C4	4224.92	68	43.9	3313.64	55	34
Apolipoprotein E	1806.58	30	68.6	2290.45	38	63.8
Ceruloplasmin	2297.77	38	37.3	2041.18	35	32
Gelsolin	1627.23	28	44.5	2018.46	32	39.6
Ig gamma-2A chain C	1826.64	30	78.6	1865.9	29	60.2
Prothrombin	1842.44	31	46	1797.2	27	39.5

Hemopexin	2202.86	38	59.6	1766.03	30	53.3
Plasma kallikrein	1128.43	19	29.9	1641.17	27	38.1
Apolipoprotein A-I	1994.88	35	71.8	1589.25	27	71
Alpha-1-antiproteinase	1918.41	30	56.4	1464.36	21	50.1
Serine protease inhibitor A3L	2020.66	32	60.5	1434.09	23	48.7
Haptoglobin	1619.4	26	46.4	1388.32	22	48.4
Vitamin D-binding protein	1697.63	25	45.6	1374.47	21	26.5
Keratin, type II cytoskeletal 5	837.78	17	22.4	1304.89	23	23.8
Plasma protease C1 inhibitor	1166.67	19	44.8	1274.7	20	38.7
Afamin	1380.53	26	36.2	1250.79	22	34.9
Ig kappa chain C region, B allele	1099.65	13	49.1	1225.27	14	56.6
T-kininogen 1	1409.3	22	40.5	1209.89	19	40.2
T-kininogen 2	1284.89	21	39.3	1156.73	18	39.1
Apolipoprotein A-IV	1490.72	25	65.7	1146.35	17	53.5
Keratin, type I cytoskeletal 10	1165.01	18	18.1	1125.03	16	18.3
Clusterin	1051.34	17	30.2	1068.36	17	34.7
Alpha-2-HS-glycoprotein	1017.69	15	46.6	1060.52	14	37.8
Serine protease inhibitor A3N	1260.76	23	54.5	1051.07	19	41.6
Serine protease inhibitor A3K	1578.19	23	51.2	1032.24	14	29.3
Ig gamma-2C chain C	887.19	15	36.5	999.59	17	36.5
Ig gamma-1 chain C	820.86	13	36.8	964.11	14	37.4
Hemoglobin subunit beta-1	894.71	13	74.8	956.58	16	85.7
Histidine-rich glycoprotein	1109.4	20	43.6	941.67	17	29.5
Kininogen-1	858.43	16	23.6	915.74	15	20.7
Keratin, type I cytoskeletal 17	551.28	10	17.8	906.79	16	25.6
C4b-binding protein alpha chain	1030.5	21	34.9	887.05	18	34.9
Hemoglobin subunit beta-2	740.05	12	73.5	871.66	14	84.4

Complement component C9	908.87	16	31.8	870.47	18	30.5
Ig gamma-2B chain C region	846.84	12	40.8	846.47	12	30.6
Inter-alpha-trypsin inhibitor heavy chain H3	1079.65	18	24	770.43	12	14.3
Coagulation factor XII	604.33	12	16.8	743.62	13	20.5
Beta-2-glycoprotein 1	678.51	13	42.8	743.5	14	44.8
Keratin, type I cytoskeletal 14	551.23	10	17.3	741.35	12	20.4
Extracellular matrix protein 1	340.37	7	19.6	732.71	12	30.2
Keratin, type II cytoskeletal 1	608.98	10	9.1	729.45	13	10.1
Transthyretin	924.3	15	71.4	713.96	12	71.4
Hemoglobin subunit alpha-1/2	749.76	11	66.2	706.96	11	66.2
Fetuin-B	663.6	10	23.5	647.79	9	18.8
Serine protease inhibitor A3M (Fragment)	738.41	11	33.5	636.26	9	22.6
Carboxylesterase 1C	830.94	13	38.1	597.07	10	22.8
Keratin, type II cytoskeletal 2 epidermal	416.93	8	8	595.19	12	6.6
Ig kappa chain C region, A allele	680.22	10	76.4	582.47	9	51.9
Keratin, type I cytoskeletal 15	451.33	7	10.3	565.06	9	10.5
Complement factor I	462.53	9	18.7	552.92	12	18.4
Keratin, type I cytoskeletal 42	286.28	6	10.6	536.81	11	15.5
Heparin cofactor 2	242.23	6	9.4	526.75	11	22.3
Ig lambda-2 chain C	521.74	8	91.3	526.72	8	72.1
Junction plakoglobin	26.98	1	1.9	491.84	9	16.9
Keratin, type I cytoskeletal 19	279.8	6	11.4	475.77	9	14.4
Apolipoprotein C-I	397.47	8	27.3	461.34	8	27.3
Glutathione peroxidase 3	394.75	7	35	456.69	8	35
Carboxypeptidase N catalytic chain	709.26	12	35.7	432.67	8	25.6
Keratin, type II cytoskeletal 75	463.93	9	11.4	427.83	8	9
Complement component C8 beta chain	470.35	11	21.2	426.61	9	19.7

Keratin, type II cytoskeletal 73	379.25	7	7.4	421.24	8	7.4
Serum amyloid P-component	465.84	9	50.4	419.42	8	38.2
Ribonuclease 4	38.81	1	25.9	414.05	8	44.2
Apolipoprotein C-IV	304.14	5	32.3	398.11	7	32.3
Angiotensinogen	376.98	6	16.1	357.56	7	19.3
Keratin, type II cytoskeletal 4	346.36	7	7.1	356.9	7	5
Platelet factor 4	182.2	3	22.9	349.19	5	38.1
Metalloproteinase inhibitor 3	106.39	3	19.9	344.85	6	29.4
Keratin, type I cytoskeletal 13	337.04	6	6.8	341.11	5	6.8
Keratin, type II cytoskeletal 1b	234.3	4	6	325.78	6	8.3
Keratin, type II cytoskeletal 6A	541.58	9	12	321.07	5	7.2
Complement C1q subcomponent subunit B	225.04	4	18.2	320.38	5	23.7
Serum paraoxonase/arylesterase 1	197.74	3	17.2	295.71	5	13
Keratin, type II cytoskeletal 8	301.96	6	9.9	291.44	6	9.9
Lipopolysaccharide-binding protein	47.23	1	2.3	289.7	5	12.1
Fibrinogen alpha chain	126.47	2	3.2	281.71	4	8.2
Corticosteroid-binding globulin	358.05	7	22.5	273.46	6	14.6
Actin, cytoplasmic 1	183.53	3	9.9	235.28	5	15.5
Carboxypeptidase B2	113.4	3	7.1	219.1	6	12.6
Vitamin K-dependent protein S	206.22	4	6.8	219.01	4	6.1
Retinol-binding protein 4	165.77	4	19.9	209.07	4	19.9
Glia-derived nexin	--	--	--	190.07	4	12.1
Complement component C6	284.42	7	10	182.55	4	4.6
Complement factor D	123.28	3	18.3	180.53	3	18.3
Complement C1q subcomponent subunit A	94.94	2	7.8	173.84	4	13.9
Complement C1s subcomponent	238.42	4	6.7	158.41	3	3.9
Ig heavy chain V region IR2	46.21	1	6.3	150.67	3	17.6

Alpha-1-acid glycoprotein	126.41	3	8.8	147.14	4	15.6
Alpha-1B-glycoprotein	93.92	2	3.5	142.7	4	6.6
Keratin, type II cytoskeletal 72	--	--	--	139.44	3	5.8
Histone H2A type 1-C	55.88	1	14.6	135.78	2	21.5
C-reactive protein	340.02	5	21.7	133.41	2	8.7
Keratin, type I cytoskeletal 20	101.39	2	4.7	130.52	2	4.7
Beta-2-microglobulin	112.7	2	19.3	121.93	2	19.3
Apolipoprotein A-II	140.95	2	17.6	119.59	2	17.6
Glyceraldehyde-3-phosphate dehydrogenase	105.7	2	11.7	118.57	3	12.9
Histone H4	35.31	1	7.8	116.77	2	21.4
Protein AMBP	160.98	4	10.3	108.32	3	8.9
C4b-binding protein beta chain	108.18	2	7.4	107.6	2	7.4
Insulin-like growth factor-binding protein complex acid labile subunit	152.45	3	5.5	107.23	2	3.5
Protein Z-dependent protease inhibitor	176.56	3	7.8	104.93	1	3.7
Mannose-binding protein A	25.73	1	2.9	102.32	3	8.8
Complement C1q subcomponent subunit C	74.73	2	7.8	99.8	2	7.8
Procollagen C-endopeptidase enhancer 1	--	--	--	99.14	1	3.6
Insulin-like growth factor-binding protein 3	--	--	--	97.05	2	6.2
Coagulation factor IX (Fragment)	144.23	3	9.9	96.3	2	6
Cystatin-C	60.51	1	12.1	87.88	1	12.1
Ubiquitin-40S ribosomal protein S27a	--	--	--	84.94	2	16
Annexin A2	--	--	--	77.71	2	6.2
Major urinary protein	48.32	1	8.8	68.45	2	13.3
Mannose-binding protein C	69.96	2	10.7	66.77	1	4.9
Histone H3.1	--	--	--	63.17	2	10.3
Phospholipase A1	58	1	2.9	57.93	1	2.9

Carboxypeptidase Q	51.54	1	1.9	57.79	1	2.3
Lumican	--	--	--	57.3	1	3.3
Peroxiredoxin-2	33.95	1	5.6	56.08	1	5.6
Insulin-like growth factor I	27.64	1	9.8	55.97	1	7.8
Cerebellin-1	38.3	1	3.6	55.2	1	3.6
Neuronal PAS domain-containing protein 4	23.93	1	1.4	53.22	2	1.4
Coagulation factor X	54.23	2	3.7	49.07	1	1.9
G patch domain-containing protein 4	26.56	1	2.2	49.01	2	2.2
Cationic trypsin-3	52.33	1	3.2	48.54	1	3.2
Phosphatidylinositol-glycan-specific phospholipase D	267.76	6	8.1	46.26	1	1.3
Attractin	49.93	1	0.7	45.94	1	0.7
Exocyst complex component 3	43.52	2	2.8	45.38	2	2.8
Adenomatous polyposis coli protein	29.48	1	0.2	45.21	2	0.6
Lysozyme C-1 GN=Lyz1 PE=1 SV=2	--	--	--	45.03	1	4.7
Selenocysteine insertion sequence-binding protein 2	51.2	2	0.8	43.8	2	0.8
Histone H2B type 1	--	--	--	41.36	1	7.2
Carbonic anhydrase 2	30.82	1	3.8	37.59	1	3.8
Apolipoprotein M	72.29	2	6.8	36.97	1	3.7
N-acylneuraminate-9-phosphatase	--	--	--	36.39	1	3.2
Complement C5 (Fragment)	--	--	--	34.71	1	19.5
SUN domain-containing ossification factor	--	--	--	34.27	1	0.7
Phospholipase A2, membrane associated	48.42	1	5.5	33.9	1	4.8
Serine/threonine-protein kinase 10	--	--	--	32.6	1	1.1
Calpain-2 catalytic subunit	28.32	1	1.1	32.42	1	1.1
Vesicle-fusing ATPase	--	--	--	31.46	1	1.2
Intraflagellar transport protein 172 homolog	31.27	1	0.7	31.21	1	0.7
Dynamin-3	20.89	1	0.8	30.84	1	0.8

Enoyl-CoA hydratase domain-containing protein 3, mitochondrial	--	--	--	30.26	1	2.3
Out at first protein homolog	--	--	--	29.27	1	4.3
Mannan-binding lectin serine protease 2	55.03	2	4.1	28.68	1	1.9
Regulated endocrine-specific protein 18	--	--	--	28.33	1	6.3
Indoleamine 2,3-dioxygenase 1	--	--	--	28.15	1	2.9
Plexin-A3	--	--	--	27.93	1	0.4
Fibronectin	335.6	7	3.5	27.01	1	0.3
Alcohol dehydrogenase [NADP(+)]	--	--	--	26.81	1	3.1
Structural maintenance of chromosomes protein 3	--	--	--	26.71	1	0.8
E3 ubiquitin-protein ligase RNF8	--	--	--	26.56	1	1.4
SAP domain-containing ribonucleoprotein	--	--	--	26.43	1	4.3
Eukaryotic translation initiation factor 3 subunit A	--	--	--	26.41	1	0.5
Zinc finger protein DZIP1L	--	--	--	26.33	1	1.7
Thioredoxin-dependent peroxide reductase, mitochondrial	--	--	--	26.08	1	2.7
Pleiotropic regulator 1	23.2	1	2.1	25.5	1	2.1
Keratin, type II cytoskeletal 80	--	--	--	25.34	1	2
Insulin-like growth factor-binding protein 5	--	--	--	25.27	1	3
Eukaryotic translation initiation factor 2-alpha kinase 1	20.29	1	1.1	25.27	1	1.1
Protein FAM228A	35.53	1	3.3	25.06	1	3.3
3-hydroxybutyrate dehydrogenase type 2	--	--	--	24.84	1	4.5
Polymerase I and transcript release factor	--	--	--	24.71	1	2.3
Cell division cycle 5-like protein	29.94	1	1.1	24.7	1	1.1
Protein wntless homolog	--	--	--	24.42	1	2.2
Leucine-rich repeat-containing protein 63	21.57	1	1.2	23.82	1	1.2
Selenoprotein P	43.04	2	8.6	23.77	1	2.9
Probable G-protein coupled receptor 158	--	--	--	23.62	1	0.7

Splicing factor, suppressor of white-apricot homolog	--	--	--	23.44	1	1.5
Microtubule-associated protein 2	--	--	--	23.37	1	0.5
Vinculin	--	--	--	23.31	1	1.2
Microtubule-associated protein 1A	--	--	--	23.21	1	0.3
Phosphorylase b kinase regulatory subunit alpha, skeletal muscle isoform	--	--	--	22.91	1	0.6
Brain-specific angiogenesis inhibitor 1-associated protein 2-like protein 1	--	--	--	22.84	1	2.9
Myosin-9	--	--	--	22.84	1	0.5
Annexin A1	21.21	1	2	22.78	1	2
Membrane-associated phosphatidylinositol transfer protein 1	--	--	--	22.61	1	0.6
Transmembrane protein 214	--	--	--	22.57	1	1
Prostaglandin E2 receptor EP4 subtype	--	--	--	22.52	1	1.8
Gastrotropin	--	--	--	22.52	1	7
Dorsal root ganglia homeobox protein	23.05	1	4.2	22.47	1	4.2
Sushi, von Willebrand factor type A, EGF and pentraxin domain-containing protein 1	--	--	--	22.45	1	0.2
Sperm-associated antigen 4 protein	23.1	1	2.9	22.28	1	2.9
Odorant-binding protein	--	--	--	22.13	1	4.7
Perilipin-1	--	--	--	22.08	1	1.7
Voltage-dependent L-type calcium channel subunit beta-2	--	--	--	21.78	1	1.1
Phosphatidylinositol 3,4,5-trisphosphate 5-phosphatase 1	--	--	--	21.6	1	0.6
Putative E3 ubiquitin-protein ligase SH3RF2	--	--	--	21.43	1	1
Proline-rich protein 5	21.92	1	1.8	21.13	1	1.8
Homeobox protein Nkx-6.1	--	--	--	21	1	2.7
Sodium-dependent serotonin transporter	--	--	--	20.92	1	1.1

Nuclear pore complex protein Nup155	--	--	--	20.9	1	0.6
Tropomyosin beta chain	--	--	--	20.86	1	3.9
Nidogen-2	--	--	--	20.72	1	0.6
Embryonic polyadenylate-binding protein 2	--	--	--	20.61	1	3.7
Cadherin EGF LAG seven-pass G-type receptor 3	--	--	--	20.6	1	0.2
Taste receptor type 1 member 1	--	--	--	20.42	1	1
P2Y purinoceptor 12	--	--	--	20.39	1	3.2
Stromelysin-3	--	--	--	20.33	1	2.2
Structural maintenance of chromosomes protein 1A	20.93	1	0.6	20.33	1	0.6
Calpain-8	--	--	--	20.26	1	1
Sodium-dependent glucose transporter 1	--	--	--	20.04	1	1.7
Nucleolar protein 16	--	--	--	20.03	1	5.1
von Willebrand factor A domain-containing protein 3A	21.86	1	0.9	--	--	--
Long-chain-fatty-acid--CoA ligase ACSBG2	21.41	1	0.9	--	--	--
Sentrin-specific protease 7	23.95	1	0.8	--	--	--
Phosphatidylinositol 4-phosphate 5-kinase type-1 alpha	47.92	2	1.3	--	--	--
Leukemia inhibitory factor receptor	47.31	1	0.7	--	--	--
Apolipoprotein C-III	26.25	1	23.8	--	--	--
A-kinase anchor protein SPHKAP	21.98	1	0.5	--	--	--
Cytochrome P450 2D4	27	1	2.4	--	--	--
Proteasome subunit alpha type-1	25.42	1	2.7	--	--	--
Choline O-acetyltransferase	24.42	1	0.9	--	--	--
60S ribosomal protein L13	24.09	1	4.3	--	--	--
Inactive pancreatic lipase-related protein 1	28.78	1	3.6	--	--	--
Phospholipase A-2-activating protein	33.94	1	1	--	--	--
Guanine nucleotide-binding protein G(o) subunit alpha	26.12	1	1.7	--	--	--
Tripartite motif-containing protein 26	21.85	1	1.3	--	--	--

Small conductance calcium-activated potassium channel protein 1	20.2	1	1.3	--	--	--
Neurofibromin	42.23	1	0.2	--	--	--
Glycosylation-dependent cell adhesion molecule 1	22.7	1	6.8	--	--	--
Unconventional myosin-Ib	35.81	1	0.4	--	--	--
Extracellular superoxide dismutase [Cu-Zn]	65.76	1	6.6	--	--	--
Geranylgeranyl transferase type-2 subunit alpha	20.79	1	1.8	--	--	--
Ligand-dependent nuclear receptor-interacting factor 1	22.69	1	0.8	--	--	--
Zinc finger RNA-binding protein	21.5	1	0.8	--	--	--
Importin subunit alpha-6	26.62	1	1.3	--	--	--
Large subunit GTPase 1 homolog	22.1	1	1.2	--	--	--
Vomeronasal type-1 receptor 98	22.11	1	2.5	--	--	--
Carboxylesterase 1E	159.9	3	11.4	--	--	--
Interleukin-1 receptor accessory protein	50.56	1	1.8	--	--	--
Zinc-alpha-2-glycoprotein	133.19	2	7.8	--	--	--
Nuclear fragile X mental retardation-interacting protein 1	32.52	1	1.2	--	--	--
Dual specificity protein phosphatase 6	24.35	1	1.6	--	--	--
Secernin-1	23.04	1	1.9	--	--	--
Transcriptional adapter 2-alpha	35.81	1	1.1	--	--	--
Probable ribosome biogenesis protein RLP24	24.57	1	4.3	--	--	--
Synaptosomal-associated protein 47	46.8	1	1.7	--	--	--
Sodium leak channel non-selective protein	33.14	1	0.3	--	--	--
Mortality factor 4-like protein 2	26.57	1	2.1	--	--	--
Apolipoprotein B-100	79.26	2	0.7	--	--	--
Kinesin-like protein KIF15	44.73	1	0.4	--	--	--
Transient receptor potential cation channel subfamily M member 7	22.34	1	0.3	--	--	--

[1] Score: number which reflects the combined scores of all observed mass spectra that can be matched to amino acid sequences within that protein. A higher score indicates a more confident match.
[2] Num. Pept: Number of peptides detected for each protein identified.
[3] Coverage: Percentage of the database protein sequence covered by matching peptide.

Table S3. Proteins identified by mass spectrometry grouped according to their molecular function using UniProt database. Only proteins detected with minimum 2 peptides were considered.

Proteins	NP-DMSA		NP-PEG-$(NH_2)_{2(2000)}$	
	Soft corona	Hard corona	Soft corona	Hard corona
**Immunoglobulins**				
• Ig kappa chain C region, B allele	x	x	x	x
• Ig kappa chain C region, A allele	-	-	x	x
• Ig gamma-1 chain C region	x	-	x	-
• Ig gamma-2B chain C region	x	x	x	x
• Ig gamma-2A chain C region	x	-	x	-
• Ig heavy chain V region IR2	-	-	x	x
• Ig gamma-2C chain C	x	x	x	x
**Lipoproteins**				
• Apolipoprotein A-IV	x	x	x	x
• Apolipoprotein E	x	x	x	x
• Clusterin	x	x	x	x
• Apolipoprotein A-I	x	x	x	x
• Apolipoprotein M	x	x	x	-
• Apolipoprotein A-II	x	x	x	x

• Apolipoprotein B-100	x	x	x	-
• Beta-2-glycoprotein 1	x	x	x	x
• Apolipoprotein C-I	x	x	x	x
• Apolipoprotein C-IV	x	x	x	x
**Coagulation**				
• Coagulation factor X	x	x	x	-
• Vitamin K-dependent protein S	-	x	x	x
• Angiotensinogen	x	x	x	x
• Fibrinogen alpha chain	-	x	-	x
• Coagulation factor IX (Fragment)	x	x	-	x
• T-kininogen 2	x	x	x	x
• T-kininogen 1	x	x	x	x
• Prothrombin	x	x	x	x
• Kininogen-1	x	x	x	x
• Heparin cofactor 2	x	x	x	x
• Coagulation factor XII	x	x	x	x
• Histidine-rich glycoprotein	x	x	x	x
• Plasma kallikrein	x	x	x	x
• Platelet factor 4	x	x	x	x
• Plasminogen	x	x	x	x
**Complement pathway**				
• Complement C1s subcomponent	-	x	-	x

• C4b-binding protein beta chain	-	x	-	x
• Complement component C9	x	x	x	x
• Plasma protease C1 inhibitor	x	x	x	x
• Complement C3	x	x	x	x
• Complement component C6	x	x	x	x
• Complement factor D	-	x	x	x
• Complement C4	x	x	x	x
• Complement factor I	x	x	x	x
• Complement component C8 beta chain	x	x	x	x
• Complement C1q subcomponent subunit C	-	x	-	x
• Complement C1q subcomponent subunit B	x	x	-	x
• Complement C1q subcomponent subunit A	-	x	x	x
• C-reactive protein	x	x	x	x
• Mannose-binding protein C	-	x	-	-
• Mannose-binding protein A	-	-	x	x
**Cellular Component**				
• Keratin, type I cytoskeletal 13	-	x	-	x
• Keratin, type I cytoskeletal 14	-	x	-	x
• Keratin, type I cytoskeletal 15	-	x	x	x
• Keratin, type I cytoskeletal 17	-	x	-	x
• Keratin, type I cytoskeletal 19	-	x	-	x
• Keratin, type I cytoskeletal 20	-	x	-	x

• Keratin, type I cytoskeletal 42	-	x	-	x
• Keratin, type II cytoskeletal 1	x	x	x	x
• Keratin, type II cytoskeletal 1b	-	x	-	x
• Keratin, type II cytoskeletal 5	-	x	-	x
• Keratin, type II cytoskeletal 4	-	x	-	x
• Keratin, type II cytoskeletal 8	-	x	-	x
• Keratin, type II cytoskeletal 2 epidermal	-	x	x	x
• Keratin, type II cytoskeletal 6A	-	x	x	x
• Keratin, type II cytoskeletal 73	-	x	-	x
• Keratin, type II cytoskeletal 75	-	x	-	x
• Actin, cytoplasmic 1	x	x	x	x
• Fibronectin	x	x	x	-
• Junction plakoglobin	-	-	-	x
• Gelsolin	x	x	x	x
**Transport**				
• Serum albumin	x	x	x	x
• Serotransferrin	x	x	x	x
• Hemopexin	x	x	x	x
• Hemoglobin subunit alpha-1/2	x	x	x	x
• Hemoglobin subunit beta-1	x	x	x	x
• Hemoglobin subunit beta-2	x	x	x	x
• Ceruloplasmin	x	x	x	x

• Haptoglobin	x	x	x	x
• Corticosteroid-binding globulin	x	x	x	x
• Exocyst complex component 3	-	x	-	x
**Enzymes**				
• Serum paraoxonase/arylesterase 1	x	x	x	x
• Carboxylesterase 1C	x	x	x	x
• Carboxylesterase 1E	-	x	x	-
• Mannan-binding lectin serine protease 2	-	x	-	-
• Carboxypeptidase Q	-	-	x	-
• Phosphatidylcholine-sterol acyltransferase	-	-	x	-
• Phosphatidylinositol-glycan-specific phospholipase D	x	x	x	-
• Glyceraldehyde-3-phosphate dehydrogenase	-	x	x	x
• Glutathione peroxidase 3	x	x	x	x
• Carboxypeptidase N catalytic chain	x	x	x	x
• Carboxypeptidase B2	x	x	x	x
• Phosphatidylinositol 4-phosphate 5-kinase type-1 alpha	-	x	-	-
• Ribonuclease 4				
	-	-	-	x
**Protease inhibitor**				
• Serine protease inhibitor A3K				
• Serine protease inhibitor A3L	x	x	x	x
• Serine protease inhibitor A3N	x	x	x	x

• Serine protease inhibitor A3M	x	x	x	x
• Alpha-2-macroglobulin	x	x	x	x
• Inter-alpha-trypsin inhibitor heavy chain H3	x	-	x	-
• Metalloproteinase inhibitor 3	x	x	x	x
• Protein Z-dependent protease inhibitor	-	x	-	x
• Glia-derived nexin	x	x	x	-
	-	-	-	x
**Nucleic acid binding**				
• Histone H2A type 1-C				
• Histone H3.1	-	-	-	x
• Histone H4	-	-	-	x
• Chromodomain-helicase-DNA-binding protein 6	-	-	-	x
• G patch domain-containing protein 4	x	-	-	-
• Neuronal PAS domain-containing protein 4	-	-	-	x
• Selenocysteine insertion sequence-binding protein 2	-	-	x	x
	-	x	-	x
**Antioxidant**	-	x	-	x
• Selenoprotein P				
	-	x	x	-
**Receptor**				
• Leukemia inhibitory factor receptor				
• Interleukin-1 receptor accessory protein	-	-	x	-
	x	-	-	-

**Other Serum components**				
• Serum amyloid P-component	x	x	x	x
• Alpha-1-acid glycoprotein	x	x	x	x
• Vitamin D-binding protein	x	x	x	x
• Murinoglobulin-1	x	x	x	x
• Retinol-binding protein 4	x	x	x	x
• Alpha-1-antiproteinase	x	x	x	x
• Alpha-1-inhibitor 3	x	x	x	x
• Transthyretin	x	x	x	x
• Protein AMBP	x	x	x	x
• Major urinary protein	x	-	x	x
• Zinc-alpha-2-glycoprotein	x	x	x	-
• Afamin	x	x	x	x
• Insulin-like growth factor-binding protein complex acid labile subunit	x	x	x	x
• Alpha-2-HS-glycoprotein	x	x	x	x
• Murinoglobulin-2	x	x	x	x
• Insulin-like growth factor-binding protein 3	-	-	-	x
• Extracellular matrix protein 1	x	x	x	x
• Alpha-1-macroglobulin	x	x	x	x
• Fetuin-B	x	x	x	x

• Alpha-1B-glycoprotein	x	x	x	x
• Attractin	x	-	x	-
• Beta-2-microglobulin	x	x	x	x
• Lipopolysaccharide-binding protein	-	-	-	x

materials

MDPI

Gel 1. 50 mM HEPES 0.1% OGP, pH 7

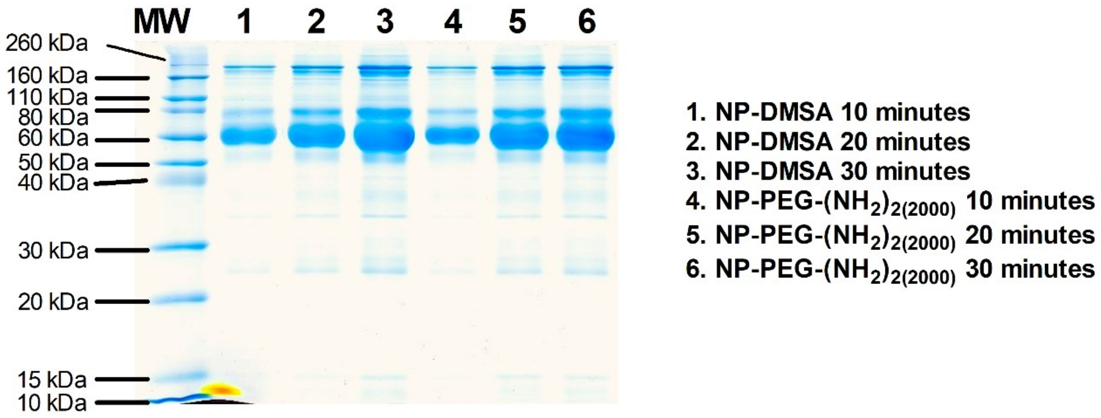

Gel 2. 100 mM NaAc 0.1% OGP, pH 5

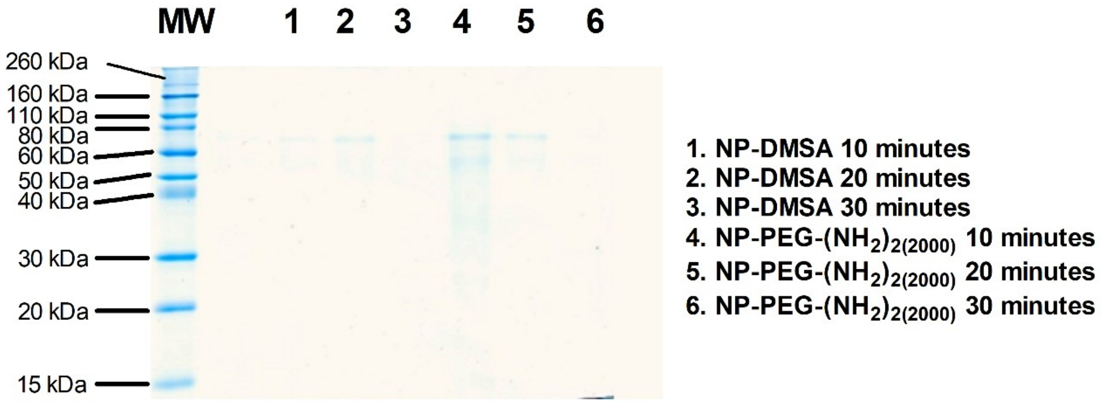

Figure S1. Coomassie blue-stained gels after electrophoresis SDS-PAGE (gel 10%). Proteins from particles incubated at 10, 20 and 30 min at 37 °C were eluted sequentially with buffers at different pH. Top panel: Proteins eluted after incubation in buffer 50 mM HEPES 0.1% OGP, pH 7 (soft corona). Lower panel: Proteins eluted after incubation in buffer 100 mM NaAc 0.1% OGP, pH 5 (hard corona).

© 2019 by the authors. Submitted for possible open access publication under the terms and conditions of the Creative Commons Attribution (CC BY) license (http://creativecommons.org/licenses/by/4.0/).
